# Assessing the Roles of Retinol, Vitamin K2, Carnitine, and Creatine in Plant-Based Diets: A Narrative Review of Nutritional Adequacy and Health Implications

**DOI:** 10.3390/nu17030525

**Published:** 2025-01-31

**Authors:** David M. Goldman, Cassandra B. Warbeck, Robby Barbaro, Cyrus Khambatta, Matthew Nagra

**Affiliations:** 1 Department of Public Health, University of Helsinki, 00014 Helsinki, Finland; 2 Department of Research and Development, Metabite Inc., New York, NY 10036, USA; 3 Department of Family Medicine, University of Alberta, Edmonton, AB T6G 2R3, Canada; cwarbeck@ualberta.ca; 4 Mastering Diabetes, Santa Monica, CA 90405, USA; robby@masteringdiabetes.org; 5 Amla Green, St. Petersburg, FL 33705, USA; cyrus@amlagreen.com; 6 Department of Family Practice, University of British Columbia, Vancouver, BC V6T 1Z3, Canada; matthew.nagra@ubc.ca

**Keywords:** nonessential nutrients, nutritional adequacy, plant-based diets, retinol, vitamin K2, carnitine, creatine, chronic disease prevention

## Abstract

Plant-based diets are associated with numerous health benefits, including reduced risks of chronic diseases. However, questions persist regarding the implications of lower dietary intakes of certain non-essential nutrients, such as retinol, vitamin K2, carnitine, and creatine, which are primarily found in animal-derived foods. This narrative review evaluates the roles of these nutrients in human physiology and examines whether their absence in plant-based diets is likely to impact health outcomes. Retinol requirements can be met through the consumption of provitamin A carotenoids in plant foods, even in individuals with reduced conversion efficiency. Endogenous synthesis adequately supports physiological needs for vitamin K2, and currently available evidence does not consistently demonstrate that dietary vitamin K2 provides additional benefits for bone or cardiovascular health. Carnitine and creatine levels may differ between individuals following omnivorous and plant-based diets, but these differences do not result in compromised muscle function, cognitive health, or metabolic outcomes. Current evidence does not indicate that the absence of these non-essential nutrients in plant-based diets adversely affects health or confers disadvantages compared to omnivorous diets.

## 1. Introduction

Plant-based diets are widely adopted for environmental, ethical, and health purposes [1]. These diets, which may include vegetarian and vegan diets, emphasize foods from plant sources and de-emphasize animal products, and have been shown to reduce the risks of chronic diseases, including cardiovascular diseases (CVDs), type 2 diabetes, and cancers, due to their higher contents of fiber, vitamins, minerals, and polyphenols and limited contents of saturated fats, heme iron, and nitrates [2]. These benefits are acknowledged in established clinical practice guidelines, many of which recommend plant-based diets for health promotion and chronic disease prevention [3]. The position statement of the Academy of Nutrition and Dietetics underscores that these diets are nutritionally adequate and appropriate for all lifecycle stages, including infancy, childhood, adolescence, pregnancy, lactation, and older adulthood, as well as for athletes [4]. However, ongoing debate centers on the nutritional adequacy of plant-based diets, particularly concerning specific nutrients that are more readily available in animal products.

Certain non-essential but biologically active nutrients, including retinol, vitamin K2, carnitine, and creatine, are less available or completely absent in plant-based diets. These nutrients play roles in key physiological processes, including vision, bone resorption, fat oxidation, and cognition [5,6,7,8]. Although humans can synthesize these nutrients from dietary precursors, concentrations achieved in individuals following plant-based diets have been hypothesized as inadequate, leading to concern that these nutritional inadequacies could diminish long-term health [9,10,11,12].

This narrative review aims to address these concerns by examining the roles of dietary retinol, vitamin K2, carnitine, and creatine in human health. The available evidence will be assessed to gauge whether the limited presence or absence of these non-essential nutrients in plant-based diets is likely to impact nutritional needs and health outcomes. By exploring current research on plant-based diet adequacy and considering practical strategies to address potential nutrient gaps, this review seeks to support informed dietary planning and improved health outcomes for individuals following plant-based diets.

## 2. Nonessential Nutrients with Potential Health Implications for Plant-Based Diets

### 2.1. Retinol

Vitamin A is an essential nutrient that promotes general growth, maintains visual function, regulates epithelial tissue differentiation, and facilitates embryonic development [5]. Deficiency can therefore result in ophthalmological, dermatological, and immune system impairments [13]. Vitamin A deficiency can affect individuals with inflammatory bowel disease and following bariatric surgery, but cases are rarely observed in developed nations due to the consumption of nutrient-rich diets [13]. Interactions with other micronutrients such as vitamin E and zinc have been observed. For example, large supplemental doses of vitamin E (500 mg) have been shown to increase the intestinal absorption and urinary excretion of vitamin A in children [14]. A consistent association between vitamin A and zinc status has not been observed in individuals residing in developed nations, although a positive association may be present in malnourished populations [15].

**Figure 1:**
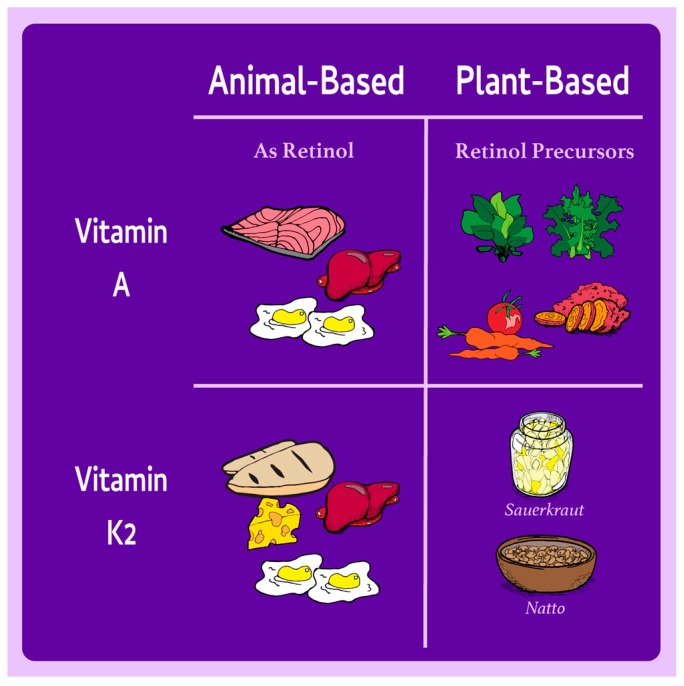
Dietary sources of vitamins A and K2 and their precursors in animal- and plant-based foods.

Vitamin A requirements can be met through the consumption of animal products, such as dairy and eggs, which provide retinol, as shown in Figure 1. Requirements can also be met with plant foods, such as orange- and yellow-colored fruits and vegetables, which provide provitamin A carotenoids such as β-carotene. Dietary retinol is more bioavailable than β-carotene. For example, the mean bioavailabilities of retinol in liver and β-carotene in vegetables have been reported to be 74% and 16%, respectively [16]. Carotenoids are converted by the β-carotene monooxygenase type 1β-carotene 15,15′-monoxygenase (*BCMO1*) enzyme in the intestine into vitamin A. Conversion ratios, which account for the bioavailability of provitamin A carotenoids and their subsequent conversion to retinol, typically are reported to range from 3.6:1 to 28:1 by weight, and differ between foods [5].

Large interindividual variability exists in vitamin A conversion efficiency, and the coefficient of variation has been reported to be as high as 221% [17]. Approximately 45% of individuals living in developed nations have been classified as “low converters” due to low postprandial conversion efficiency following supplementation, which is measured by the retinyl ester/β-carotene ratio in the chylomicron fraction [17]. The degree of impairment varies, with in vivo estimates indicating a 32–69% reduction in the conversion of carotenoids to retinol, depending on the genetic variant [18]. One case report described a genetic variant that reduced carotenoid oxygenase activity by 90%, resulting in mild hypovitaminosis A and necessitating supplementation, but such cases are notably rare [19]. Under a more typically impaired conversion rate, such as a 32% reduction in capacity in individuals with a single genetic variant (379 V) affecting *BCMO1* activity, 200 g (one cup) of cooked orange sweet potato supplies enough β-carotene (96.7 mcg/g) to produce enough retinol to surpass the Recommended Daily Allowance of 900 and 700 mcg/day for men and women of all ages, respectively [5,20]. Individuals with both *BCMO1* genetic variants (267 S + 379 V) and 69% impairment in conversion would surpass requirements by consuming 400 g (two cups) of cooked orange sweet potato per day [5,20]. This suggests that vitamin A requirements can be achieved, even in individuals with lower conversion efficiency, through modest intakes of commonly consumed and readily available plant foods. This appears valid regardless of background dietary pattern, especially considering that additional dietary sources of carotenoids are commonly consumed in quantities that contribute further to β-carotene intakes [21].

Concerns have been expressed that a lack of dietary retinol may be particularly problematic for populations following plant-based diets, who must rely on the endogenous production of vitamin A from carotenoid precursors [9,10,12]. However, research has found similar serum retinol concentrations across dietary patterns, with levels of 2.5, 2.2, and 2.1 µmol/L in individuals following omnivorous, vegetarians, and vegan diets, respectively [22]. Each of these values exceeds the vitamin A deficiency cut-off of <0.7 µmol/L.

Individuals following plant-based diets can meet vitamin A requirements by eating carotenoid-rich foods like dark green leafy vegetables such as spinach, and orange and yellow fruits and vegetables such as sweet potatoes, carrots, and tomatoes, as shown in Figure 1. Pairing these foods with small quantities of fat-rich foods (~5 g fat/meal), including avocadoes, nuts, and seeds, can enhance carotenoid absorption and make conversion to retinol more efficient [23]. The results of a recent systematic review indicate that regular consumption of carotenoid-rich foods, such as orange-fleshed sweet potatoes, can improve vitamin A status in individuals with marginal levels, highlighting the practical impact of these strategies in preventing and correcting deficiency [24]. Individuals with reduced conversion efficiency may therefore benefit from additional emphasis on carotenoid-rich foods, although intakes consistently exceed requirements in populations following plant-based diets [22].

### 2.2. Vitamin K2

Vitamin K is an essential nutrient widely known for its role in blood clotting and increasingly recognized for its potential contributions to cardiovascular and bone health [8]. The two main forms of vitamin K are vitamin K1 (phylloquinone) and vitamin K2 (multiple menaquinones). Vitamin K1 is found in green leafy vegetables and other photosynthetic organisms and constitutes the majority of dietary vitamin K intake, but demonstrates lower bioavailability and a shorter half-life than vitamin K2 [25,26,27,28]. Vitamin K2 is produced by bacteria such as *Bacillus subtilis*, *Saccharomyces cerevisiae*, and *S. coelicolor* through a complex process that involves many metabolic pathways, including glycolysis, the hexose monophosphate shunt, the shikimate pathway, the methyl-D-erythritol 4-phosphate or mevalonate pathway, and the futalosine pathway [29]. Vitamin K2 exists in several subtypes, labeled MK-*n* (menaquinone-*n*), based on the number of isoprene units in their side chains. Dietary sources of vitamin K2 include fermented plant foods such as natto and animal products such as meat, certain cheeses, and liver [26,27]. Vitamin K intake can influence the effects of anticoagulants such as warfarin. It has therefore been recommended that patients taking these medications maintain consistent vitamin K intakes in order to decrease intrapatient variability in anticoagulation responses and increase therapeutic safety [30]. Dietary sources of vitamin K2 are shown in Figure 1.

Among the menaquinones, MK-4 and MK-7 are the most extensively researched. MK-4 is found in animal products such as meat, eggs, and liver, but does not reliably increase serum levels unless given in supplemental doses far exceeding typical dietary intakes [28]. This is because MK-4 is primarily synthesized endogenously from vitamin K1 by the UbiA prenyltransferase domaining containing 1 (*UBIAD1*) enzyme in extrahepatic tissues [31,32]. Animal modeling suggests that significant interindividual variability in endogenous synthesis may exist due to genetic and metabolic factors [33]. In contrast, MK-7 sourced from fermented plant foods, such as natto, reliably increases serum levels and remains biologically active for up to 144 h, compared to approximately 24 h of activity for MK-4 [27,28]. These differences in bioavailability and bioactivity highlight the potential significance of dietary MK-7 from fermented plant foods.

Vitamin K2 has demonstrated benefits for bone health through its activation of osteocalcin, a protein that facilitates calcium incorporation into the bone matrix. Interventional trials in postmenopausal women provide supplements containing 45 mg/day of MK-4 and typically find that supplementation results in elevated concentrations of activated osteocalcin, increased bone mineral density, and reduced risk of fractures [34,35,36,37]. Supplementation with 180 μg/day of MK-7 has also been shown to ameliorate the age-related loss of bone mass in postmenopausal women [38]. Although these findings suggest that MK-4 and MK-7 supplementation can improve bone health and reduce fracture risk in postmenopausal women, research has found no significant effect on bone mineral density in men [39].

Vitamin K2 has also been studied in relation to cardiovascular health. It has shown potential benefits by activating matrix Gla-protein, a potent inhibitor of vascular calcification [40,41]. Observational studies have found associations between higher vitamin K2 intake and reduced CVD risk. For example, a study of 53,372 Danish citizens found that participants with the highest intakes of vitamin K2 had a 14% lower risk of CVD-related hospitalizations compared to those with the lowest intakes during the 21 years of follow-up [42]. Similarly, the Prospect-European Prospective Investigation into Cancer and Nutrition (Prospect-EPIC) cohort study of 16,057 women demonstrated an inverse association between vitamin K2 intake and CVD risk [43]. However, in both studies, the final models were adjusted for fatty acid intake. These adjustments may have resulted in vitamin K2 intake serving as a proxy for cheese consumption rather than other foods high in saturated fat such as meat. This is consequential because dairy fat intake has been shown to result in smaller increases in LDL-cholesterol and CVD risk compared to other sources of animal fat [44,45]. These findings may therefore reflect the benefits of substituting dairy fat for other animal fat sources rather than protective effects of vitamin K2 intake on CVD risk. Caution is therefore warranted in interpreting the results of these studies, especially considering the low bioavailability of MK-4 demonstrated in interventional research [28].

Preliminary research has also investigated the effects of vitamin K2 supplementation on cardiovascular function in healthy individuals. McFarlin et al. (2017) conducted a randomized controlled trial to explore the effects of eight weeks of supplementation with 150–300 mg/day of MK-7 on cardiac output in 26 active individuals [46]. The results showed that vitamin K2 supplementation was associated with a 12% improvement in maximal cardiac output (*p* = 0.031), which the authors attributed to increased heart rate rather than stroke volume. These intake levels significantly exceed dietary provisions, limiting the application of these findings in omnivorous versus plant-based dietary contexts. Nonetheless, additional research on MK-7 supplementation should be conducted to extend the findings beyond cardiovascular function to hard exercise performance outcomes.

Plant-based diets, which are naturally high in vitamin K1, provide adequate amounts to meet clotting-related needs and may support endogenous MK-4 synthesis (Kim et al., 2019). There is a lack of evidence to suggest that the absence of dietary K2 from animal products negatively impacts health outcomes. Plant-based diets are associated with favorable cardiovascular outcomes, likely due to their overall nutrient profiles, which include abundant fruits, vegetables, and other whole foods [47]. Fermented plant-based foods, such as natto, serve as effective dietary sources of K2. When additional intake is desired, plant-derived supplements, such as MK-7, provide a reliable means of enhancing K2 status as opposed to most animal-derived products, which do not provide highly bioavailable forms of vitamin K2 [48]. Research into the effects of MK-4 and MK-7 supplementation on measures of bone health and cardiovascular disease in individuals following plant-based diets would further elucidate the potential of vitamin K2 to influence key outcomes.

### 2.3. Carnitine

**Figure 2:**
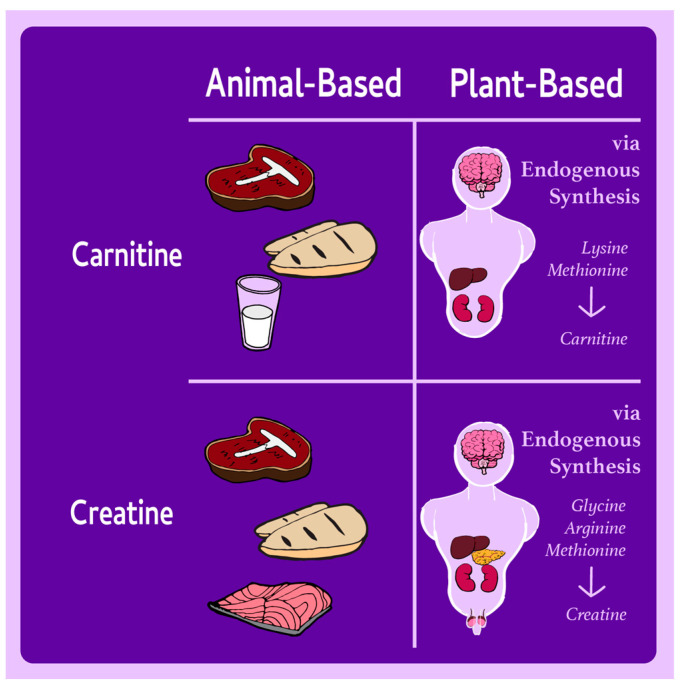
Sources of carnitine and creatine in animal- and plant-based diets.

Carnitine is a nonessential amino acid that is integral to fatty acid metabolism and adenosine triphosphate (ATP) production, and plays key roles in cellular detoxification, cell membrane stabilization, gluconeogenesis, and ketogenesis [7]. It is primarily stored in skeletal muscle tissue, which houses more than 95% of total bodily stores, due to its central role in energy metabolism [49]. Carnitine is synthesized in the liver, kidneys, and brain from the essential amino acids lysine and methionine [50], as shown in Figure 2. This process involves protein-bound lysine, which is enzymatically methylated to form trimethyllysine during protein synthesis. Trimethyllysine then undergoes four enzymatic reactions to produce carnitine. Dietary carnitine intake and excretion do not affect endogenous synthesis, which is approximately 14.4 mg/day, an amount that is sufficient to meet the needs of healthy people [51]. However, carnitine biosynthesis rates vary between individuals. A person weighing 70 kg is estimated to synthesize 11–34 mg/day of carnitine [52]. Carnitine reabsorption by the kidneys is 95% efficient, sustaining adequate levels and preventing deficiency in generally healthy individuals, including those following plant-based diets [52]. Primary carnitine deficiency occurs in up to 5 in 10,000 people due to a genetic defect in the carnitine receptor, and secondary deficiencies can result from renal failure, liver disease, or nutritional deficits in lysine, methionine, or metabolic cofactors [50].

Diet can also make significant contributions to carnitine levels, with provisions varying widely between foods and dietary patterns. The most concentrated dietary source of carnitine is red meat (91 mg/100 g of beef), and smaller amounts are present in poultry, fish, and dairy products [53,54]. With the exception of mushrooms (which contain 2.77–7.02 mg/100 g of carnitine), plant foods contain trace amounts of carnitine (<0.1 mg/100 g) [54]. Omnivorous diets supply approximately 23–135 mg/day of carnitine, whereas plant-based diets provide approximately 1 mg/day [53]. This contrast in dietary carnitine content has led to speculation that plant-based diets increase the risk of carnitine deficiency [10,55].

Observational research demonstrates that carnitine levels differ between individuals following omnivorous and plant-based diets. Compared to individuals following omnivorous diets, those following plant-based diets maintain total and free plasma carnitine concentrations that are 17–36% and 14–34% lower, respectively [56,57,58,59]. This occurs despite renal adaptations that decrease carnitine excretion and increase resorption in individuals following plant-based diets [57,59]. However, it has been suggested that these small differences in plasma carnitine do not represent nutritionally significant differences in carnitine status [57].

Skeletal muscle carnitine concentrations in individuals following omnivorous versus plant-based diets have demonstrated equivocal findings [49,56]. The provision of 2 g/day of supplemental carnitine for 12 weeks did not influence skeletal muscle function or energy metabolism in young individuals eating plant-based diets [56]. Additional investigation of longer durations and in older individuals is therefore required to determine whether such differences in muscle carnitine content exert any meaningful effects on musculoskeletal function [49]. Mixed findings on the effectiveness of carnitine supplementation for athletic performance in omnivorous individuals introduce additional questions pertaining to the potential ergogenicity of the amino acid [60].

Research has also examined the effects of carnitine supplementation for weight management, cognitive function, and cardiovascular health. Although carnitine is necessary for fatty acid oxidation, a meta-analysis including 37 randomized controlled trials found that the effects of carnitine supplementation on weight loss (weighted mean difference (WMD) = −1.21 kg, 95% confidence interval (CI), −1.73, −0.68; *p* < 0.001) and body mass index (BMI) (WMD = −0.24 kg/m^2^, 95% CI, −0.37, −0.10; *p* = 0.001) were modest, and no significant changes in body composition or waist circumference were observed [61]. The relationship between carnitine supplementation and cognitive function was investigated in a Cochrane systematic review including 16 trials, which found no evidence of benefits on objective assessments of dementia, and concluded that evidence to recommend the routine use of carnitine in clinical practice is lacking [62]. The effects of carnitine supplementation on cardiovascular health were explored in a meta-analysis including 13 controlled trials, which found that carnitine supplementation was associated with significant reductions in the risks of angina (risk ratio [RR], 0.60; 95% CI, 0.50–0.72; *p* < 0.00001), ventricular arrhythmias (RR, 0.35; 95% CI, 0.21–0.58; *p* < 0.0001), and all-cause mortality (odds ratio [OR], 0.73; 95% CI, 0.54–0.99; *p* = 0.05; RR, 0.78; 95% CI, 0.60–1.00; *p* = 0.05), but not myocardial reinfarction or heart failure [63]. However, this research has been criticized for underestimating the risk of bias and for presenting effect sizes that are much greater than those found in well-studied interventions [64]. In addition, a more recent double-blind, randomized, placebo-controlled, two-center trial including 157 patients with metabolic syndrome found that carnitine supplementation produced greater increases in total and LDL-cholesterol, as well as 9.3% greater carotid atherosclerotic stenosis compared to placebo [65]. Mendelian randomization research has also demonstrated an association between genetically predicted L-carnitine levels and higher risk of coronary artery disease (OR, 1.07 per standard deviation (SD) increase in L-carnitine, 95% CI, 1.02, 1.11) [66]. However, these results did not reach significance after statistical correction, indicating the possibility of a chance finding.

Overall dietary patterns may influence body weight, cognitive function, and CVD risk to a greater extent than the presence or absence of dietary carnitine. Meta-analytic research with a mean follow-up duration of 14 years has demonstrated lower mean BMI in individuals following plant-based versus omnivorous diets [67]. Associations between plant-based diets and dementia risk have shown either protective or neutral associations [68,69], whereas differences in cognitive outcomes have not been observed [70,71]. This discrepancy may be influenced by differences in the quality of the plant-based diets studied, specifically the degree to which whole and refined plant-based foods are included [72]. Long-term adherence to plant-based dietary patterns has also been associated with reduced risks of CVD incidence and mortality [73,74]. These findings suggest that the benefits of plant-based diets outweigh any theoretical detriments of inadequate carnitine intake on weight management, cognitive function, and CVD risk. Empirical support for carnitine insufficiency in individuals following plant-based diets is therefore required to demonstrate that an absence of dietary carnitine confers increased risks of adverse health outcomes.

### 2.4. Creatine

Creatine is an organic compound synthesized mainly in the kidneys, liver, and pancreas, but also in the brain and testes [75], as shown in Figure 2. Creatine supports the rapid recycling of ATP from adenosine diphosphate (ADP) and is critical for energy-demanding processes such as cognitive performance and muscle contraction [6]. The amino acids arginine, glycine, and S-adenosyl-L methionine are used to form creatine, which is subsequently converted into phosphocreatine by the creatine kinase enzyme. This enzyme facilitates the reversible transfer of a phosphoryl group from ATP to creatine, allowing cells to efficiently store and regenerate energy as needed. Creatine biosynthesis is consistent, although nephrectomy and creatine supplementation have been shown to reduce synthetic rates [76]. Aging does appear to reduce creatine synthesis [77], although research in animal models has indicated that long-term social isolation and circadian rhythm disruptions may downregulate production [78,79]. Creatine deficiency disorders are characterized by inborn errors related to creatine metabolism and transport. Although these disorders have been reported in <300 individuals, clinical manifestations include developmental delay, cognitive dysfunction, and speech-language disorders, with oral creatine supplementation foundational to treatment [80].

Dietary creatine is concentrated in meat and fish, as shown in Figure 2, and is present in small amounts in dairy, yielding ~1 g/day in the diets of individuals following omnivorous diets [81]. Creatine is absent in plants, which contributes to lower serum and muscle creatine concentrations in individuals following plant-based diets [58,82,83,84,85,86,87]. Despite significant interindividual variability in brain creatine levels in populations following plant-based and omnivorous diets, mean concentrations remain comparable between diet groups, likely due to endogenous synthesis in the brain [82,88,89]. Furthermore, creatine uptake at the blood–brain barrier may be limited by the lack of SLC6A8 transporters, which reduces the reliance of the brain on circulating creatine [90]. As a result, supplemental creatine in doses higher than typical dietary intake may be necessary to increase brain creatine concentrations [90].

There is no evidence that plant-based diets increase the risk of cognitive impairment. Iguacel et al. (2020) conducted a systematic review and meta-analysis of cross-sectional, prospective, and interventional studies that included 17,809 individuals and found no significant association between plant-based diets and cognitive impairment in studies of short-term (<1 year) or long-term (≥1 year) follow-up durations [70]. This conclusion is supported by Gatto et al. (2021), who studied 132 healthy older community-dwelling participants in the Adventist Health Study-2 cohort, 20% of whom reported lifelong abstention from meat and fish, and found no significant differences in processing speed, executive function, or memory or language abilities between individuals following omnivorous or plant-based diets. The study also found that a more stable dietary pattern over the life course, a characteristic observed more consistently in individuals following plant-based diets, was associated with better memory and language abilities [71]. If the absence of dietary creatine in plant-based diets impaired cognitive function, such deficits would be apparent in these studies. Instead, the evidence suggests that endogenous creatine synthesis sufficiently supports cognitive health in populations consuming plant-based diets.

Studies investigating creatine supplementation do not consistently show greater cognitive improvements in individuals following plant-based versus omnivorous diets. Sandkühler et al. (2023) conducted a randomized, placebo-controlled, cross-over trial to determine the effects of six weeks of creatine supplementation (5 g/day) on cognitive performance in 123 participants following omnivorous or plant-based diets. Creatine supplementation produced a small beneficial effect on cognitive outcomes in both groups, although significant differences between groups were not observed [89]. In contrast, Benton and Donohoe (2011) conducted a randomized, placebo-controlled trial in which 128 adult females consumed 20 g/day of creatine or placebo for five days. Creatine supplementation did not affect verbal fluency or vigilance, but mitigated the decline in memory in individuals following plant-based, but not omnivorous diets. Reduced response variability in a choice reaction-time task was observed across both diet groups [91]. The differing doses used in these studies may be responsible for the divergent findings. It has been hypothesized that the modest creatine contents of omnivorous diets, and moderate doses provided in common supplemental regimens, do not influence cognition [89]. Instead, higher supplemental doses may be required to exert an effect [90]. This may result from endogenous creatine synthesis within the brain creating resistance to exogenous creatine sources [88,92]. These findings indicate that individuals following plant-based diets do not consistently experience greater cognitive benefits from creatine supplementation compared to those following omnivorous diets. If dietary creatine deficiencies significantly limited cognitive function, supplementation would predictably yield greater improvements in populations consuming plant-based diets. The lack of such consistent outcomes suggests that dietary creatine is not a critical determinant of cognitive function.

The absence of creatine in plant-based diets has also been questioned for its potential impact on lean body mass and physical performance. While individuals following plant-based diets generally exhibit lower muscle creatine levels, this has not been shown to impair body composition, muscle strength, muscle function, or exercise capacity in young, middle-aged, or older adults [93,94,95,96]. Creatine supplementation reliably enhances strength and hypertrophy in individuals following omnivorous and plant-based diets [97], and its effects may be potentiated through co-ingestion with carbohydrates or protein [90], but evidence linking dietary creatine to these outcomes is limited [98]. The physiological effects of supplementation are more pronounced in older versus younger individuals [82], but do not consistently differ between diet groups [97], suggesting that dietary creatine is not influential for physical performance [98]. Therefore, evidence to suggest that the absence of creatine in plant-based diets results in cognitive or physical performance deficits is lacking. A summary of key findings for creatine and other aforementioned non-essential nutrients can be found in Table 1.

## 3. Conclusions

The nutritional adequacy of plant-based diets continues to be a topic of scientific inquiry, particularly concerning non-essential but physiologically significant nutrients such as retinol, vitamin K2, carnitine, and creatine. Although these nutrients are abundant in animal-derived foods, evidence presented in this narrative review suggests that their absence in plant-based diets is unlikely to detract from health. Moreover, there is a lack of evidence to suggest that the presence of these nutrients in animal foods translates to improved health outcomes in individuals following omnivorous versus plant-based diets. Future research should investigate the role of endogenous synthesis and bioavailability of these nutrients in individuals following plant-based diets, and the roles of dietary and supplemental forms of these non-essential nutrients on long-term health outcomes. This review highlights the lack of scientific evidence indicating that the absence of dietary retinol, vitamin K2, carnitine, and creatine in plant-based diets is deleterious to human health. Empirical evidence is therefore required before recommendations to ingest these non-essential nutrients through animal foods can be justified.

From health policy and clinical practice perspectives, promoting plant-based diets as nutritionally adequate and aligned with chronic disease prevention goals is essential. Policymakers can support public health by emphasizing evidence-based strategies to optimize nutrient bioavailability, such as consuming beta-carotene-rich vegetables paired with small amounts of healthy fats to enhance vitamin A status. Fortified foods and plant-derived supplements can also be promoted to address specific nutrient needs. Healthcare providers play a critical role in tailoring plant-based dietary recommendations to individual needs, such as advising patients with reduced carotenoid-to-retinol conversion efficiency to incorporate additional servings of beta-carotene-rich foods or recommending creatine supplementation for athletes engaging in high-intensity activities. For populations at risk, such as postmenopausal women, clinicians can suggest vitamin K2 supplementation to support bone health. Combining evidence-based policy initiatives with personalized clinical guidance can support individuals to adopt diverse, nutrient-dense plant-based diets while addressing unique nutritional concerns, ultimately advancing public health outcomes.

## Figures and Tables

**Table 1:** Summary of Key Findings on Non-Essential Nutrients in Plant-Based Diets.

Nutrient	Primary Roles in the Body	Dietary Sources in Omnivorous Diets	Dietary Sources in Plant-Based Diets	Health Implications
Retinol	Vision, immune function, cell differentiation, embryonic development	Liver, eggs, dairy	Provitamin A carotenoids (e.g., carrots, sweet potatoes, spinach), which can be paired with fat for enhanced absorption	Conversion efficiency varies, but adequate intakes can meet requirements in plant-based diets, even for low converters.
Vitamin K2	Bone health (activates osteocalcin), cardiovascular health (inhibits vascular calcification)	Animal products (MK-4), fermented dairy	Natto (fermented soy), plant-derived MK-7 supplements	MK-4 is poorly bioavailable; plant-based sources like MK-7 are effective and reliable for supplementation if needed.
Carnitine	Fatty acid metabolism, energy production, detoxification	Red meat, poultry, fish	Trace amounts in mushrooms; synthesized endogenously from lysine and methionine	No evidence of deficiency in plant-based diets; endogenous synthesis is sufficient in healthy individuals.
Creatine	Rapid ATP recycling, cognitive function, muscle contraction	Meat, fish	None; synthesized endogenously from glycine, arginine, and methionine	Lower muscle stores in plant-based dieters, but no adverse impact on health. Supplementation can benefit high-performance populations if needed.

## Data Availability

No new data were created or analyzed in this review. Data sharing is not applicable to this article.

## Abbreviations

The following abbreviations are used in this manuscript:

ADP	Adenosine diphosphate
ATP	Adenosine triphosphate
BCMO1	β-carotene 15,15′-monoxygenase
BMI	Body mass index
CVD	Cardiovascular diseases
CI	Confidence interval
MK-4	Menaquinone-4
MK-7	Menaquinone-7
RR	Risk ratio
SD	Standard deviation
UBIAD1	UbiA prenyltransferase domain containing 1
WMD	Weighted mean difference
